# A path planning approach for mobile robots using short and safe Q-learning

**DOI:** 10.1371/journal.pone.0275100

**Published:** 2022-09-26

**Authors:** He Du, Bing Hao, Jianshuo Zhao, Jiamin Zhang, Qi Wang, Qi Yuan

**Affiliations:** 1 College of Computer and Control Engineering, Qiqihar University, Qiqihar, China; 2 College of Telecommunication and Electronic Engineering, Qiqihar University, Qiqihar, China; Nottingham Trent University School of Science and Technology, UNITED KINGDOM

## Abstract

Path planning is a major challenging problem for mobile robots, as the robot is required to reach the target position from the starting position while simultaneously avoiding conflicts with obstacles. This paper refers to a novel method as short and safe Q-learning to alleviate the short and safe path planning task of mobile robots. To solve the slow convergence of Q-learning, the artificial potential field is utilized to avoid random exploration and provides a priori knowledge of the environment for mobile robots. Furthermore, to speed up the convergence of the Q-learning and reduce the computing time, a dynamic reward is proposed to facilitate the mobile robot towards the target point. The experiments are divided into two parts: short and safe path planning. The mobile robot can reach the target with the optimal path length in short path planning, and away from obstacles in safe path planning. Experiments compared with the state-of-the-art algorithm demonstrate the effectiveness and practicality of the proposed approach. Concluded, the path length, computing time and turning angle of SSQL is increased by 2.83%, 23.98% and 7.98% in short path planning, 3.64%, 23.42% and 12.61% in safe path planning compared with classical Q-learning. Furthermore, the SSQL outperforms other optimization algorithms with shorter path length and smaller turning angles.

## Introduction

Path planning is the calculation of a feasible path from a start node to a goal node in a map or grid without colliding with obstacles on the way [1]. It requires mobile robots (MRs) to be equipped with sensors, onboard computers, and motion systems to plan and move. Many current algorithms can implement path planning for MR. The former approach includes search-based planning algorithms: A* [2], D* [3], etc. A* algorithm is one of the popular heuristic algorithms [4], which is widely used in the field of path optimization. Its unique feature is that it introduces global information when checking each possible node in the shortest path, estimates the distance between the current node and the end point. Derived from the traversal A*, D* algorithm repairs the existing search information instead of reconstructing the whole search graph [5, 6]. It is suitable for dynamic environment compared to A*. Due to their universality and ease of implementation, these algorithms achieve significant results in searching for paths, but the search time grows exponentially with the resolution size and search depth of the map; Sampling-based planning algorithms: rapidly random-exploring tree (RRT) [7], probabilistic roadmap (PRM) [8], etc. The advantage of these algorithms is that they are effective and fast in high-dimensional path search, and the disadvantage is that these algorithms usually sample the environment for random search to find paths, the results are often not optimal and it is difficult to find a feasible path in environments with narrow passages. Artificial potential field (APF) [9] and BUG algorithm [10] are widely used in path planning for local obstacle avoidance. Although these algorithms are computationally simple for dynamic obstacle environments and fast in path search, the optimal path is often not obtained, and the search path may be erroneous when the obstacles are large in a complex environment. Another type of algorithm is the intelligent algorithms: it is an algorithm that people model by nature-inspired or human mind to imitate solving problems [11]. Typical algorithms are particle swarm optimization (PSO) [12], ant colony optimization (ACO) [13], and other algorithms. Intelligent algorithms play an effective role in solving complex dynamic environments, but there are common problems such as slow computation speed, poor stability, poor real-time performance, and easy to fall into local optimality.

Path planning can be divided into global and local path planning depending on the acquisition of the environment [14]. Global path planning is to plan a path for MR in a completely known environment. The location and shape of obstacles in the environment are known to the MR. The global path planning is often optimal or sub-optimal. As the robot travels along the global path, if there are obstacles in the global path that are not modeled in the known environments, the robot will collide with the obstacles. This requires local path planning in completely unknown or partially known environments. The local planning method integrates the modeling of the environment with the search, which provides real-time feedback and correction of the planning results. However, the planning results may not be optimal due to the lack of global environmental information. The two planning methods need to work together to allow the robot to better plan its travel path from the start to the target position.

To solve global and local path planning problems, a common approach is to hybridize two algorithms [15–18]. In [19], the authors proposed a method using the A* algorithm for global path planning, and the APF for local path planning in unknown areas. It provides the best solutions in less time when obtaining optimal path distance. [20] proposed an improved ACO for a globally optimal path, and an improved APF is subsequently employed to avoid unknown obstacles during navigation. The authors in [21] proposed an improved PSO for global path planning and APF for local dynamic obstacle avoidance, to solve the local minimum problem. The two algorithms are combined to solve the MR global and local path planning problem, avoiding the defects of a single algorithm that is prone to get trapped in a local minimum [22].

All of the above-mentioned algorithms have their superiorities and limitations. Most of the researches are environment-based precise modeling and positioning navigation. However, the real environments are partially or completely unknown. It requires MR evasion of unknown static and dynamic obstacles. Moreover, most of the papers treat MR as a mass point to plan the path, resulting in the path being too close to the obstacle, which leads the MR to collide with obstacles [23].

To solve the defects of the above path planning method, the proposed approach enables the mobile robot to plan the path in two modes: short and safe path planning. The effectiveness, superiority, and rapidity of the SSQL algorithm are demonstrated through simulation and comparison experiments. The SSQL algorithm proposed in this paper is applied to solve the path planning for MRs with the following main contributions:

1. The SSQL is a novel proposal that can find short and safe condition for MR path planning, outperforming motion planning proposals based on the state-of-the-art algorithms.
2. Two path planning schemes are proposed, one is to plan the shortest path length (Proximity to obstacles), and the other is to plan the safe path length (away from obstacles) from the starting point to the target point.
3. This paper hybridizes Q-learning with APF to initialize Q-table, avoiding the random movement around the start point at the beginning of the algorithm.
4. The proposed algorithm changes the constant reward into a combination of static and dynamic reward; speeds up the convergence of the algorithm and avoids the algorithm falling into a dead-end path blocked by obstacles.
5. By comparing with other algorithms, it is proved that the proposed algorithm in this paper can solve the MR path planning problems for two modes: short and safe path planning.

## Materials and methods

In this section, this paper first describes the MR path planning problem, then outlines classical Q-learning, and at last proposes the SSQL algorithm.

### Problem formulation

**Fig 1:**
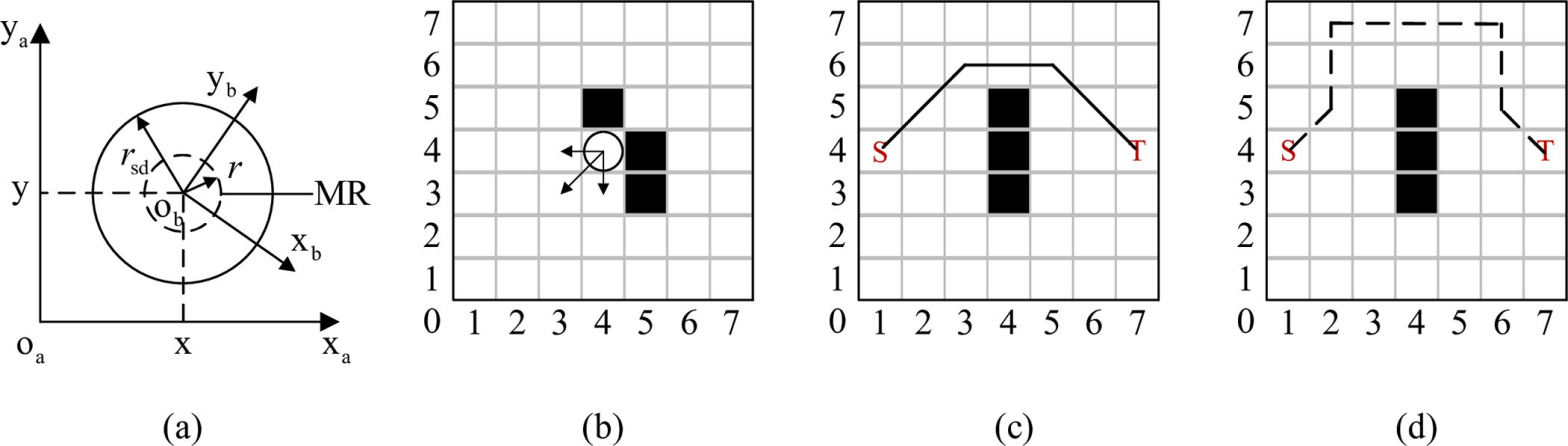
Path planning problem formulation: (a) Schematic diagram of MR; (b) Direction of motion when there is an obstacle around the MR; (c) Short path length condition; (d) Safe path length condition. (Circle for MR; white grid for *S*_free_; black grid for *S*_obs_; solid black line is the path planned by SSQL).

To better illustrate the planning problem using SSQL in this paper, path planning process is shown in Fig 1. The schematic diagram of MR and the definition of safe distance are shown in Fig 1A, where, x_a_ and y_a_ represent the earth-fixed frame, x_b_ and y_b_ represent the body-fixed frame of MR, *r* represents the radius of the circular MR. It is assumed that the origin of the body-fixed frame is located in the MR centre of mass. To plan the path away from obstacles, this paper defines the MR safety radius as *r*_sd_, to avoid collision of the planned path with obstacles. For better obstacle avoidance, this paper defines: when there are no obstacles around MR can move to the adjacent eight directions (north, northeast, east, southeast, south, southwest, west and northwest).

MR cannot cross the obstacles barrier diagonally. When the obstacles are located around the MR, the movement directions of MR are shown in Fig 1B. In a Cartesian coordinate, the MR aims to plan a feasible path from the start S (1,4) to the target T (7,4) without collision with obstacles. Fig 1C and 1D show two models of planning path for a MR. Fig 1C is the short path length mode, the MR can reach the target from the start with the short path length. Fig 1D is the safe condition mode, the MR always distance from obstacle 2*r*_sd_ planning path.

### Q-learning algorithm

Q-learning [24] is a value-based reinforcement learning algorithm proposed by Watkins in 1989. MR path planning can be expressed as follows: at each discrete time series (off-line strategy temporal-difference) [25]. The MR interacts with the environment through continuous feedback, generating more data (states and returns) and using the new data to further improve its own behavior. Q-table is the expectation that *Q*(*s*_*t*_,*a*_*t*_) can gain by taking action *a*_*t*_ in state *s*_*t*_, updated by the Formula shown in Formula 1.


$$Q(s_{t},a_{t})\leftarrow (1-\alpha )*Q(s_{t},a_{t})+\alpha *[r_{t+1}+\gamma \underset{a}{\max}Q(s_{t+1},a_{t+1})]$$ (1)

The above function can also be written as Formula 2 and Formula 3:

$$Q(s_{t},a_{t})\leftarrow (1-\alpha )*Q(s_{t},a_{t})+\alpha *[r_{t+1}+\gamma V(s_{t+1})]$$ (2)

$$V(s_{t})\leftarrow V(s_{t})+\alpha *[r_{t+1}+\gamma V(s_{t+1})-V(s_{t})]$$ (3)

where, *α* (0≤*α*≤1) is a learning rate parameter, *γ* (0≤*γ*≤1) is a discount rate parameter.

In this paper, the Q-learning is applied to MR path planning for the following reasons:

1. Reinforcement learning has good interaction with the environment without the need for positive or negative labels [26]. MR gains current knowledge by exploring and learning from the environment, and improve their operational strategies to adapt to the environment.
2. The Q-learning algorithm is highly exploratory and is an iterative trial-and-error process, where multiple attempts are made for each possible pair of state actions to obtain the optimal policy as long as time allows.
3. Q-learning uses off-policy [27], the selection of actions according to the target strategy can be used to control the distance between MR and the obstacle.

### The proposed short and safe Q-learning algorithm

In this section, the proposed SSQL algorithm is elaborated in four stages for solving the short and safe path planning. To better describe the studied content, the MR path planning problem discussed in this paper has the following premises:

1. The MR and the obstacle environment are three-dimensional objects in practice. To simplify the problem, this paper treats their motion space as two-dimensional coordinates.
2. The MR path planning studied in this paper is divided into two parts: short and safe path length. The location, shape and size of obstacles in the environment are known in the path planning.
3. The task of the MR is to reach the target from the start with the shortest and safe path length.

### Q-table initialization

Classical Q-learning usually initializes the Q-table to zero or normally distributed random numbers, which leads the MR to choose actions randomly in the exploration phase. Resulting in slow convergence and long computing time of the algorithm. To optimize the problem, this paper combines APF with Q-learning to optimize the initial Q-table. The reasons are: the APF can be combined with the Q-learning both in global and local path planning; it is easy to implement in grid maps and provide prior knowledge of the environment for MR; to speed up convergence and reduce computing time.

The information in the environment (starting point, target point, obstacle shape, size and location coordinates) is completely known for MR, so Coulomb’s law is used to model the APF for the grid environment, as shown in Formula 4, Formula 5 and Formula 6:

$$U_{a}(s_{t})=\frac{1}{2}k_{a}\rho_{g}^{2}(s_{t})$$ (4)

$$U_{r}(s_{t})=\{\begin{array}{l} \frac{1}{2}k_{r}{\left(\frac{1}{\rho_{\mathrm{ob}}(s_{t})}-\frac{1}{\rho_{o}}\right)}^{2},\rho_{\mathrm{ob}}(s_{t})\leq \rho_{o}\\ 0\;,\rho_{\mathrm{ob}}(s_{t})\leq \rho_{o} \end{array}$$ (5)

$$U_{n}(s_{t})=U_{r}(s_{t})+U_{a}(s_{t})$$ (6)

$$U(s_{t})=|\frac{U_{\max}-U_{n}(s_{t})}{U_{\max}}|$$ (7)

$$Q_{0}(s_{0},a_{0})=U(s_{t})$$ (8)

where, *U*_a_(*s*_*t*_) is the gravitational field producing gravitational force in state *s*_*t*_; *U*_*r*_(*s*_*t*_) is the gravitational field producing gravitational force in state *s*_*t*_; *U*_*n*_(*s*_*t*_) is the total potential energy of state *s*_*t*_; *ρ*_*g*_(*s*_*t*_) is the Euclidean distance between state *s*_*t*_ and the center of the target point; *ρ*_ob_(*s*_*t*_) is the Euclidean distance between state *s*_*t*_ and the center of the obstacle; *ρ*_o_ is the obstacle influence factor; *k*_a_ and *k*_*r*_ are the scale factors; *U*(*s*_*t*_) is the potential energy in state *s*_*t*_; *U*_max_ is the highest potential energy in state *s*_t_

### Action selection

To avoid obstacles and reach the target point with the short path length, eight directions of movement are defined for the MR (north, northeast, east, southeast, south, southwest, west and northwest). When the safe distance of MR is defined as *r*_sd_ = 5m, the corresponding movement and movement distance are:

action1: *a*_1_ = move north 10m;

action2: $a_{2}=\mathrm{move}\;\mathrm{northeast}\;10\sqrt{2}m$;

action3; *a*_3_ = move east 10m;

action4: $a_{4}=\mathrm{move}\;\mathrm{southeast}\;10\sqrt{2}m$;

action5: *a*_5_ = move south 10m;

action6: $a_{6}=\mathrm{move}\;\mathrm{southwest}\;10\sqrt{2}m$;

action7: *a*_7_ = move west 10m;

action8: $a_{8}=\mathrm{move}\;\mathrm{northwest}\;10\sqrt{2}m$

### Reward function

To solve this problem, a combined static and dynamic reward function is proposed in the DMQL algorithm, which provides the target and current position as a priori knowledge to the MR. When the MR is closer to the target position, the larger the reward obtained, prompting it to move to the target position and speed up the convergence.

In this paper, two different sets of reward functions are set for the two working modes of SSQL. The reward functions of the short path length are shown in Formulas 9 ~ 13. The planned path is shown in Fig 1C.

$$reward=r_{s}*(1+r_{d})$$ (9)

$$r_{s}=\{\begin{array}{l} 1,\;\mathrm{hor}.\;\mathrm{or}\;\mathrm{ver}.\;\mathrm{movement}\\ 2,\;s_{t+1}\;\mathrm{is}\;\mathrm{the}\;\mathrm{start}\;\mathrm{node}\\ 1/\sqrt{2},\;\mathrm{diagonal}\;\mathrm{movement}\\ 10,\;s_{t+1}\;\mathrm{is}\;\mathrm{the}\;\mathrm{target}\;\mathrm{node}\\ -\inf,\;s_{t+1}\;\mathrm{is}\;\mathrm{the}\;\mathrm{forbidden}\;\mathrm{node} \end{array}$$ (10)

$$d_{t}=\sqrt{{\left(y_{\mathrm{target}}-y_{t}\right)}^{2}+{\left(x_{\mathrm{target}}-x_{t}\right)}^{2}}$$ (11)

$$d_{t+1}=\sqrt{{\left(y_{\mathrm{target}}-y_{t+1}\right)}^{2}+{\left(x_{\mathrm{target}}-x_{t+1}\right)}^{2}}$$ (12)

$$r_{d}=\frac{d_{t}-d_{t+1}}{\left|d_{t}-d_{t+1}\right|}$$ (13)

where, *r*_s_ is the static reward; *r*_*d*_ is the dynamic reward; *d*_*t*_ is the Manhattan distance from the target position in the state *s*_*t*_; *d*_*t*+1_ is the Euclidean distance from the target position in the next state *s*_*t*+1_; (*x*_*t*_,*y*_*t*_) is the coordinate in the state *s*_*t*_; (*x*_*t*+1_,*y*_*t*+1_) is the coordinate in the state *s*_*t*+1_; (*x*_target_,*y*_target_) is the coordinate in the target position.

The reward functions of the safe path length are the same with Formula 9, Formula 11, Formula 12 and Formula 13. Formula 10 is modified as Formula 14. The planned path is shown in Fig 1D. By setting such a reward function the distance to the obstacle can be controlled.


$$r_{s}=\{\begin{array}{l} 1,\;\mathrm{hor}.\;\mathrm{or}\;\mathrm{ver}.\;\mathrm{movement}\\ 2,\;s_{t+1}\;\mathrm{is}\;\mathrm{the}\;\mathrm{start}\;\mathrm{node}\\ 1/\sqrt{2},\;\mathrm{diagonal}\;\mathrm{movement}\\ 10,\;s_{t+1}\;\mathrm{is}\;\mathrm{the}\;\mathrm{target}\;\mathrm{node}\\ -\inf,\;\mathrm{the}\;\mathrm{eight}\;\mathrm{grids}\;\mathrm{around}\;s_{t+1}\\ \;\mathrm{are}\;\mathrm{the}\;\mathrm{forbidden}\;\mathrm{nodes} \end{array}$$ (14)

### Short and safe condition path planning

In this paper, the proposed SSQL algorithm is applied to the path planning problem of MR, and its basic ideas are as follows: the APF is used to initialize Q-table, to provide the prior knowledge in the environment to the MR. The static and dynamic reward are combined to optimize the reward function, to induce the MR to move toward the target point and control the distance between MR and obstacles.

To sum up, the flow of MR using SSQL algorithm is as follows: when MR is located at the starting point, select the mode (short or safe condition path planning). When considering MRs driving as far away from shoreline objects as possible to avoid collisions with reefs, the safe condition is used. When distance and energy consumption are taken into account, short condition path planning is used to path the shortest path length for MR. The combination of the two models allows for better path planning for MRs based on actual situations.

## Results and discussion

It is difficult to apply the SSQL algorithm directly to the path planning problem of MR, and it requires repeated training to obtain the optimal action strategy, therefore, this paper demonstrates the performance and generality of the SSQL through several numerical simulations in this section.

### Environments

**Fig 2:**
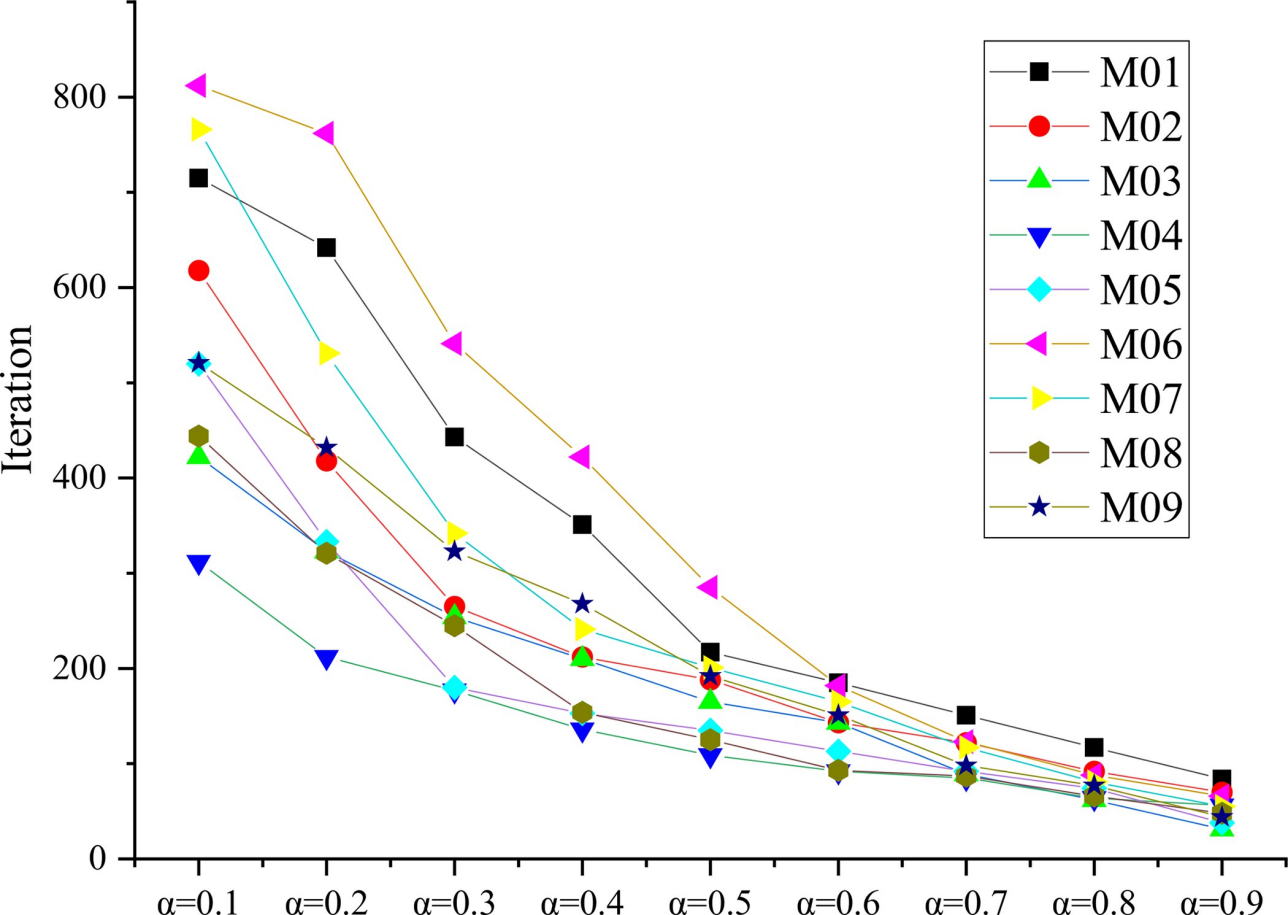
The number of iterations that path length converges to the optimum.

This paper considers nine different environment maps for MR to validate our approach. Each map is defined as a square grid environment with sides of length 20*2*r* (*r* is the radius of MR). The simulation maps are represented by M01 to M09 as shown in Fig 2. The center of each grid is marked by a Cartesian coordinate system, with the x-axis indicating the horizontal direction and the y-axis indicating the vertical direction. The coordinates are expressed as (*x*,*y*). The first and second dimensions of the grid map represent the x-horizontal and y-vertical coordinates, respectively.

### Performance metrics

To test the effectiveness, safety, and speed of the proposed SSQL algorithm in a comprehensive and concrete way, the MR path is evaluated in three performance metrics: path length (Formula 15), turning angle (Formula 19), and computing time.

### Path length (m)


$$Path\;Length(m)=\sum_{i=0}^{n}\sqrt{{\left(y_{i+1}-y_{i}\right)}^{2}+{\left(x_{i+1}-x_{i}\right)}^{2}}$$ (15)

where, *i* = 0,1,2,…,*n*, when *i* = 0, the MR is at the starting position *S* = (*x*_*o*_,*y*_*o*_), when *i* =n, the MR is at the target position *T* = (*x*_*n*_,*y*_*n*_), (*x*_*i*_,*y*_*i*_) represents the coordinates of the current state of the MR; (*x*_*i*+1_,*y*_*i*+1_) represents the coordinates of the next state of the MR.

### Turning angle (rad)

The turning angle is the sum of the change in heading angle of MR from the start to the target. When the turning angle is smaller, the path is smoother, less energy is consumed, and the completion time of the mission is shorter. This paper defines the turning angle of MR from the start to the target position as shown in Formula 16 ~ Formula 19.

$$a_{i}=\sqrt{{\left(y_{i}-y_{i-1}\right)}^{2}+{\left(x_{i}-x_{i-1}\right)}^{2}}$$ (16)

$$b_{i}=\sqrt{{\left(y_{i+1}-y_{i}\right)}^{2}+{\left(x_{i+1}-x_{i}\right)}^{2}}$$ (17)

$$c_{i}=\sqrt{{\left(y_{i+1}-y_{i-1}\right)}^{2}+{\left(x_{i+1}-x_{i-1}\right)}^{2}}$$ (18)

$$Angle(\mathrm{rad})=\sum_{i=1}^{n}\left(\pi -ar\cos\frac{a_{i}^{2}+b_{i}^{2}-c_{i}^{2}}{2*a_{i}*b_{i}}\right)$$ (19)

where, *i* = 0,1,2,…,*n*, (*x*_*i*_,*y*_*i*_) represents the coordinates of the current state of MR; (*x*_*i*+1_,*y*_*i*+1_) represents the coordinates of the next state of MR.

### Computing time (s)

Computing time is consumed by the algorithm through iterative computation is denoted as *Time*(s). The average value of 30 repeated tests in this paper is taken as the computing time. The shorter computing time, the less waiting time for MR to execute the action.

### Parameters selection

Before the SSQL algorithm is executed, the values of the two parameters: learning rate *α*(0≤*α*≤1) and decay rate *γ*(0≤*γ*≤1) in the Q-value update Formula 1 need to be determined. According to Watkins et al. [24], when the *α* value is small, the agent goes through all states in the environment and calculates all possible actions, and the Q value converges to the optimal value. When the *γ* value is large, it can expand the exploration range of the agent and prevent the agent from falling into the problem of local optimum. Therefore, based on the above theory, when *γ* = 0.9, this paper records the number of iterations that path length converges to the optimum (Fig 2). Repeat the test 30 times to take the average value, considering the computing time of MR, both *α* and *γ* are taken as 0.9 [23].

To evaluate the generality and universality of the SSQL algorithm applied to the MR path planning problem, the SSQL algorithm is compared with CQL (Classical Q-learning), PSO (Particle Swarm Optimization), GWO (Grey Wolf Optimization) [28], DA [29] and MFO (Moth-Flame Optimization) [30] in different simulation environments, respectively. PSO [31] is the basic path planning comparison algorithm. It originates from the study of bird flock predation behavior, which is characterized by easy implementation; GWO is inspired by the leadership hierarchy and hunting mechanism of the gray wolf in nature, which has the characteristics of strong convergence performance and few parameters; DA and MFO are the state-of-the-art algorithms. DA is inspired by the static and dynamic swarming behaviors of dragonflies in nature, which has the advantages of strong stability, good search speed, and global searchability, etc. MFO simulates the special navigation mechanism of moths using lateral positioning during night flight, which has the performance characteristics of strong parallel optimization ability, global superiority, and not easy to fall into local minima. To test the SSQL algorithm proposed in this paper under the same conditions, the parameter settings of the compared algorithms are shown in Table 1.

**Table 1:** Parameters setting of DFQL, CQL, PSO, GWO, DA, and MFO.

Algorithms	Parameters Selection	*Max*	*Pop*
DFQL	*α* = 0.9, *γ* = 0.9, *ρ*_*o*_ = 2, *k*_a_ = 1.5, *k*_*r*_ = 1.5	100	—
CQL	*α* = 0.9, *γ* = 0.9		—
PSO	*c*_1_ = 2, *c*_2_ = 2		30
	$\omega =\omega_{\max}-Iter*((\omega_{\max}-\omega_{\min})/Max)$		
	*ω*_max_ = 0.9, *ω*_min_ = 0.2		
GWO	*a* = 2−Iter*(2/*Max*), *C* = 2*rand()		
DA	a=2*rand()*(0.1−0.1*Iter/(Max/2))		
	c=2*rand()*(0.1−0.1*Iter/(Max/2))		
	e=0.1−0.1*Iter(Max/2)		
	*f* = 2*rand()		
	*r* = 3+(*Iter*/*Max*)		
	s=2*rand()*(0.1−0.1*Iter/(Max/2))		
	$\omega =\omega_{\max}-Iter*((\omega_{\max}-\omega_{\min})/Max)$		
	*ω*_max_ = 0.9, *ω*_min_ = 0.2		
MFO	t=(−1+Iter*(−1/Max)−1)*rand()+1, *b* = 0.9		

where, *Max* is the maximum number of iterations, *Pop* is the population size; PSO: *c*_1_ and *c*_2_ are the learning factor, *ω* is the linearly decreasing weight (LDW) (Tian et al., 2018), *ω*_max_ is the maximum inertia weight, *ω*_min_ is the minimum inertia weight; GWO: *a* is a constant, the initial value is 2, and decreases linearly from 2 to 0 with the iteration of the algorithm, *C* is a random number between 0 and 2; DA: *a* is the alignment weight, *c* is the cohesion weight, *e* is the natural enemy weight factor, *f* is the prey weight factor, *r* is the neighborhood radius, and *s* is the separation weight; MFO: *t* is the path coefficient, *b* is the logarithmic spiral shape constant; rand() denotes a random number between [0,1], and *Iter* denotes the current iteration number.

### Short path planning for mobile robot

**Fig 3:**
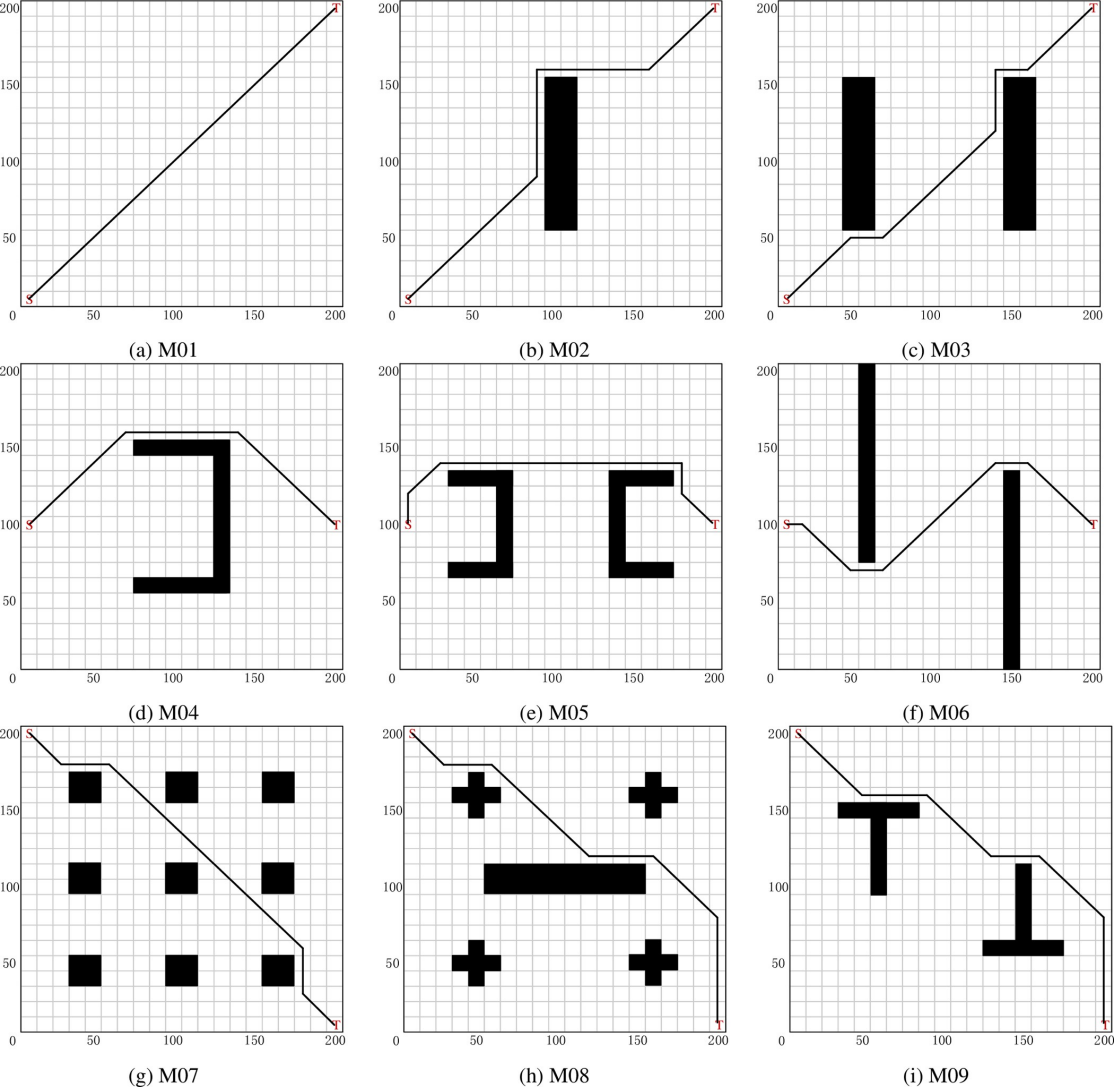
Short path planning (solution) for MR in different test environments (Each map shows the best path obtained by the SSQL algorithm, S is the starting point and T is the target point).

In this section, a short path planning for MR using the SSQL is proposed. The proposed algorithm enables the MR to perform the task with the shortest or shorter path length from the starting point to the target point. To verify the effectiveness and generalizability of the proposed algorithm, tests were conducted on the nine grip maps in Fig 3. Each map shows the best path obtained by the SSQL algorithm (black solid line), S is the starting point and T is the target point.

**Fig 4:**
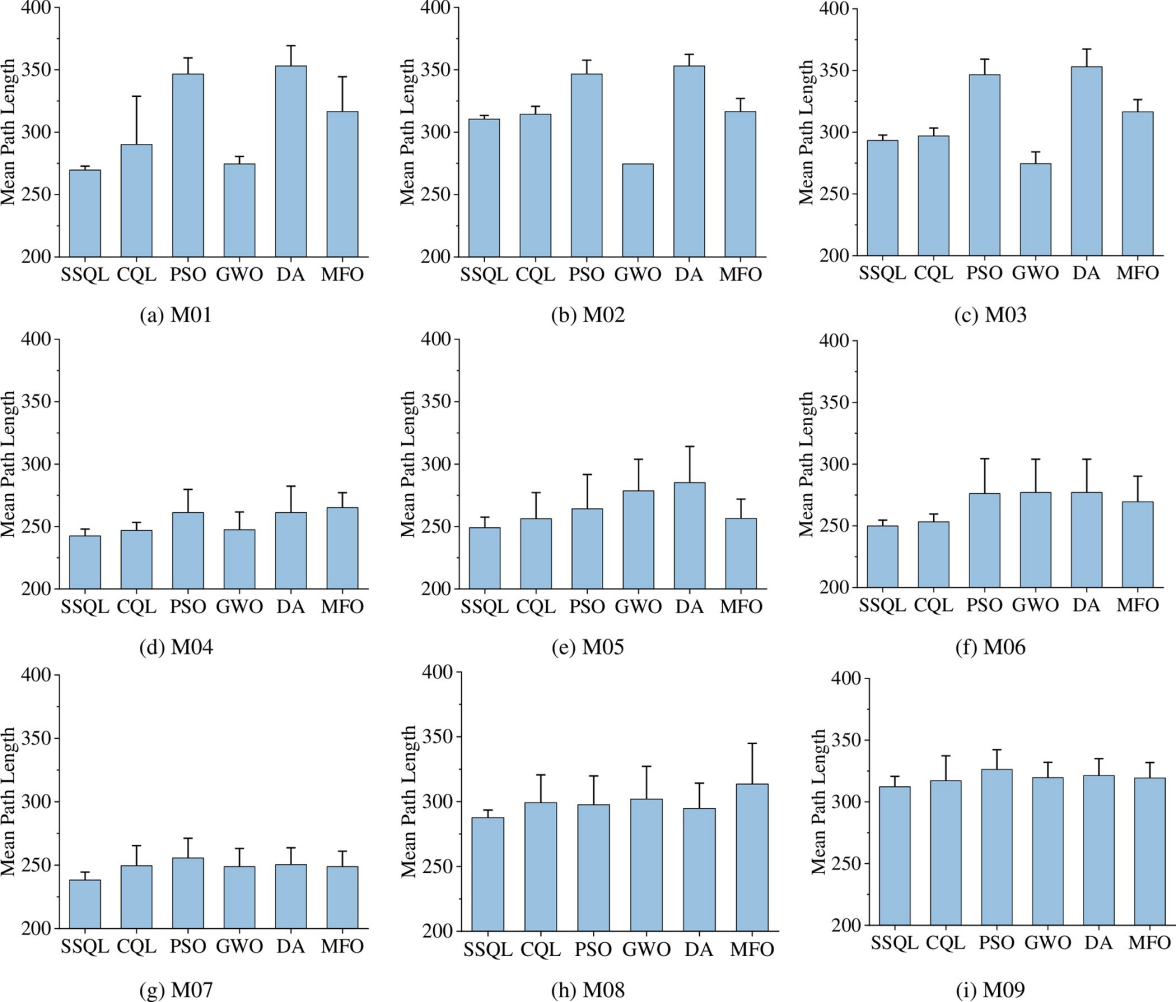
Compared mean path length of various path planning algorithms for MR (The interval on each bar denotes the standard deviation of the path length).

The optimal (suboptimal or optimal under optimal conditions) results of SSQL in 30 repeated tests (the path with the smaller turning angle and computing time under the same path length condition is taken) are shown in Fig 3 (the paths of other compared algorithms are not indicated in Fig 3 for a clearer representation of the paths of SSQL). Experimental data and analysis results were recorded in Table 2, and check whether there is a significant difference between SSQL algorithm and other comparison algorithms by *t*-test (considering 0.05 significance level). Compared mean path length of various path planning algorithms for MR are shown in Fig 4.

**Table 2:** A comparison between SSQL, CQL, PSO, GWO, DA and MFO for short path planning. The lowest (best) both mean, standard deviation, and the values of path length bigger than the level 0.05 of significance are highlighted.

Env	Statistics		SSQL	CQL	PSO	GWO	DA	MFO
M01	Path Length	Mean	**269.68**	290.18	346.61	274.56	353.05	316.54
			Std. Dev.	**3.11**	38.59	12.88	5.96	16.32	27.86
			*t*-test	-	6.99e-3	5.54e-26	2.57e-4	2.21e-23	3.74e-10
		Angle	Mean	**0.10**	0.43	0.63	0.54	0.75	0.66
			Std. Dev.	0.26	0.44	0.19	0.42	0.28	**0.24**
		Time	Mean	5.92	6.54	**2.76**	2.92	3.67	2.77
M02	Path Length	Mean	310.49	314.39	317.52	**309.71**	315.56	313.81
		Std. Dev.	2.97	6.36	11.13	**0.00**	9.36	10.45
		*t*-test	-	2.15e-3	**3.28e-1**	1.52e-6	2.92e-7	**4.73e-1**
	Angle	Mean	1.10	1.66	**0.81**	0.77	0.86	0.84
		Std. Dev.	**0.12**	**0.12**	0.14	0.06	0.21	0.17
	Time	Mean	5.21	5.79	3.32	3.37	4.06	**3.05**
M03	Path Length	Mean	**293.30**	297.01	308.14	299.94	327.87	309.12
		Std. Dev.	**4.46**	6.36	12.59	9.52	14.38	9.89
		*t*-test	-	1.16e-2	5.20e-7	1.28e-3	1.89e-14	7.80e-10
	Angle	Mean	1.62	1.72	1.25	1.36	**1.01**	1.22
		Std. Dev.	0.16	0.17	0.17	0.17	0.32	**0.13**
	Time	Mean	6.09	6.97	3.46	3.62	4.55	**3.31**
M04	Path Length	Mean	**242.47**	246.88	261.22	247.43	261.22	265.08
		Std. Dev.	**5.47**	6.42	18.51	14.24	21.13	12.08
		*t*-test	-	5.82e-3	6.54e-6	**8.284e-2**	4.41e-5	1.21e-11
	Angle	Mean	**0.58**	0.73	0.79	0.72	0.72	0.79
		Std. Dev.	**0.15**	0.24	0.22	0.18	0.16	0.24
	Time	Mean	7.24	8.19	4.24	**2.98**	3.64	3.17
M05	Path Length	Mean	**249.04**	256.22	264.21	278.58	285.20	256.50
		Std. Dev.	**8.47**	20.98	27.49	25.33	28.96	15.45
		*t*-test	-	**9.05e-2**	6.64e-3	**8.28e-2**	4.41e-5	1.21e-11
	Angle	Mean	**1.33**	1.42	1.65	1.63	1.53	1.71
		Std. Dev.	**0.18**	0.19	0.23	0.28	0.34	0.35
	Time	Mean	6.17	6.51	3.78	3.88	4.21	**3.59**
M06	Path Length	Mean	**249.93**	253.25	276.18	277.02	277.02	269.56
		Std. Dev.	**4.72**	6.35	28.26	26.98	26.98	20.71
		*t*-test	-	2.54e-2	2.10e-5	6.64e-6	6.64e-6	1.65e-5
	Angle	Mean	1.37	1.57	1.82	1.83	1.88	1.94
		Std. Dev.	**0.27**	0.33	0.62	0.47	0.46	0.41
	Time	Mean	5.39	5.79	4.33	**4.12**	4.56	4.71
M07	Path Length	Mean	**238.23**	249.55	255.63	248.73	250.40	248.74
		Std. Dev.	**6.31**	15.79	15.55	14.38	13.28	12.24
		*t*-test	-	7.90e-4	1.53e-6	7.27e-4	4.88e-5	1.38e-4
	Angle	Mean	**1.05**	1.58	1.58	1.52	1.51	1.50
		Std. Dev.	**0.15**	0.55	0.49	0.38	0.43	0.44
	Time	Mean	6.18	6.52	3.49	3.91	4.66	**3.34**
M08	Path Length	Mean	**287.61**	299.19	297.54	301.95	294.78	313.55
		Std. Dev.	**5.88**	21.41	22.26	25.27	19.50	31.35
		*t*-test	-	7.32e-3	2.41e-2	4.82e-3	**6.22e-2**	1.02e-4
	Angle	Mean	**1.33**	1.51	1.85	1.53	1.68	1.68
		Std. Dev.	**0.16**	0.32	0.68	0.34	0.56	0.48
	Time	Mean	6.94	7.51	**3.35**	3.92	3.51	3.47
M09	Path Length	Mean	**312.19**	317.16	326.26	319.64	321.30	319.37
		Std. Dev.	**8.46**	20.11	15.96	12.37	13.67	12.50
		*t*-test	-	**2.01e-1**	7.84e-5	6.60e-3	2.41e-3	9.17e-3
	Angle	Mean	**1.30**	1.52	1.83	1.84	1.78	1.79
		Std. Dev.	**0.17**	0.43	0.52	0.53	0.60	0.53
	Time	Mean	6.15	6.35	**3.31**	3.97	4.28	4.12

### Safe path planning for mobile robot

In this section, a safe planning for MR using the SSQL is proposed. The proposed algorithm enables the MR to perform the task with the safe path length (keep away from obstacles) from the starting point to the target point. To verify the effectiveness and generalizability of the proposed algorithm, tests were conducted on the nine grip maps in Fig 3. Since the path planned by the comparison algorithms (PSO, GWO, DA and MFO) cannot move away from the obstacles, it usually adopts the way of expanding the obstacles to achieve the purpose of planning the safe path. In this paper, the obstacles are expanded by one grid (1x1 expanded to 3x3 grids) to plan the path of the comparison algorithms. Each map shows the best path obtained by the SSQL algorithm (black solid line), S is the starting point and T is the target point.

**Fig 5:**
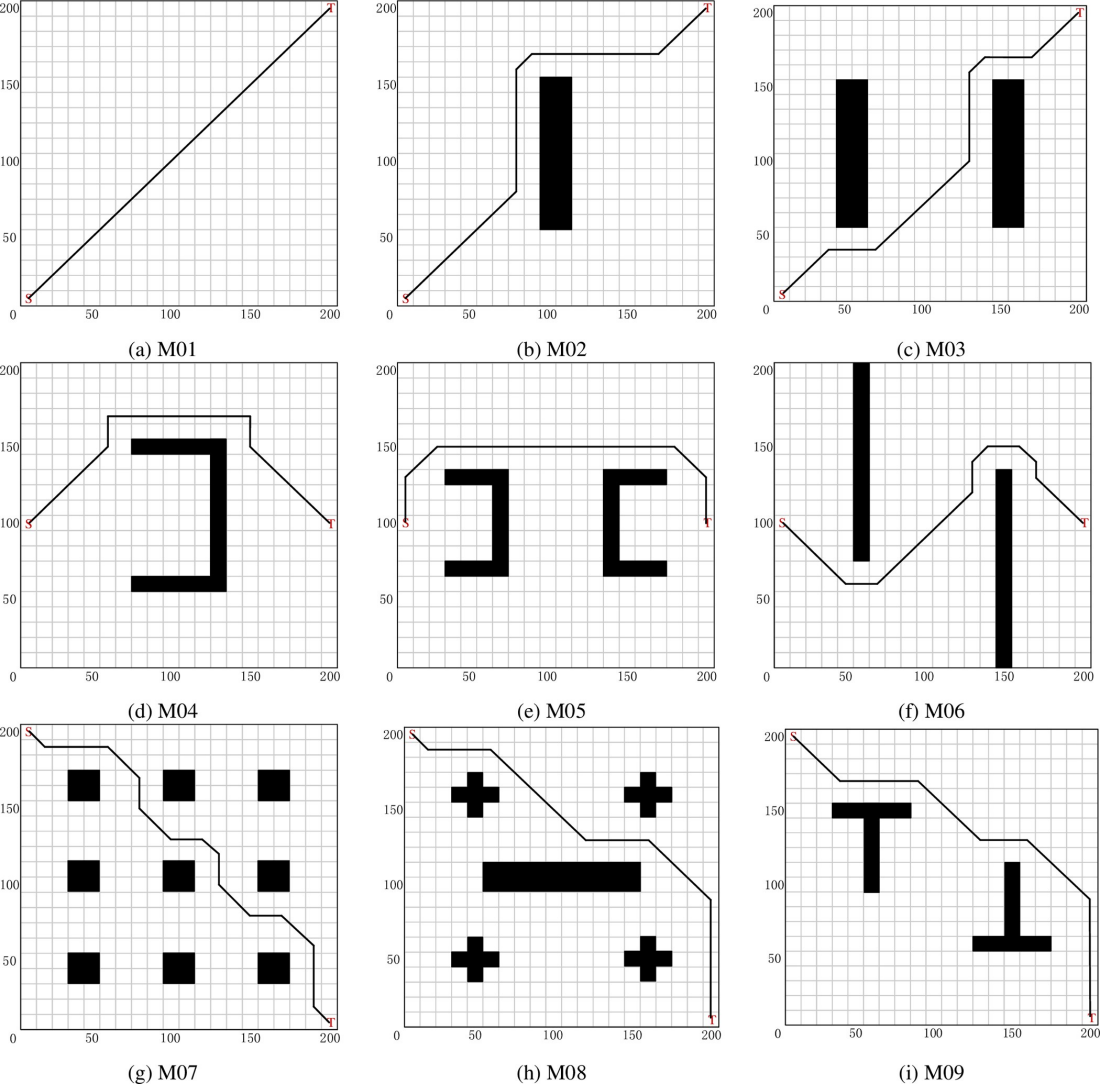
Safe path planning (solution) for MR in different test environments (Each map shows the best path obtained by the SSQL algorithm, S is the starting point and T is the target point).

The optimal (suboptimal or optimal under optimal conditions) results of SSQL in 30 repeated tests (the path with the smaller turning angle and computing time under the same path length condition is taken) are shown in Fig 4 (the paths of other compared algorithms are not indicated in Fig 5 for a clearer representation of the paths of SSQL).

**Fig 6:**
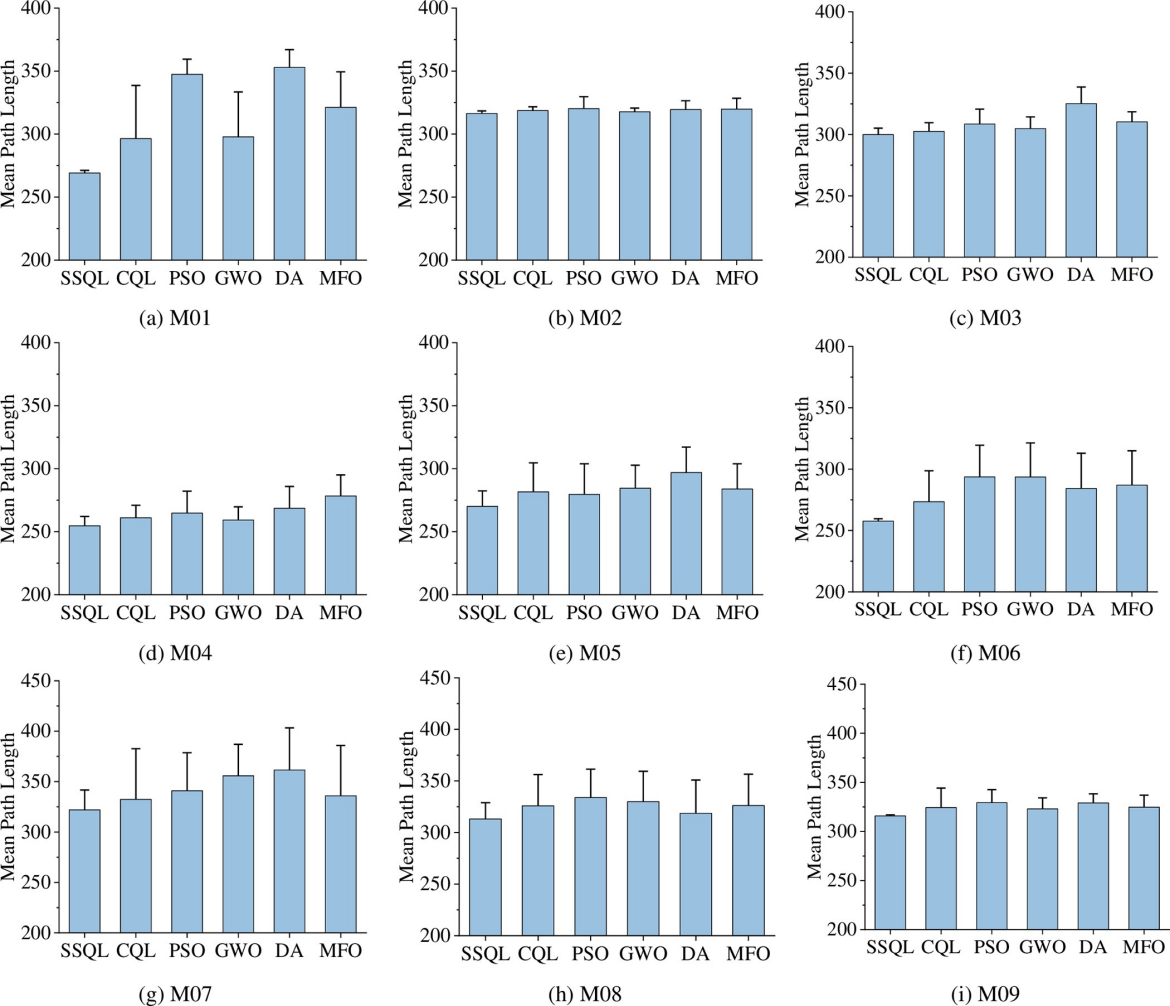
Compared mean path length of various path planning algorithms for MR (The interval on each bar denotes the standard deviation of the path length).

Experimental data and analysis results were recorded in Table 3, and check whether there is a significant difference between SSQL algorithm and other comparison algorithms by *t*-test (considering 0.05 significance level). Compared mean path length of various path planning algorithms for MR are shown in Fig 6.

**Table 3:** A comparison between SSQL, CQL, PSO, GWO, DA and MFO for safe path planning. The lowest (best) both mean, standard deviation, and the values of path length bigger than the level 0.05 of significance are highlighted.

Env	Statistics		SSQL	CQL	PSO	GWO	DA	MFO
M01	Path Length	Mean	**269.09**	296.43	347.39	297.79	352.86	321.23
		Std. Dev.	**2.14**	42.16	11.98	35.66	14.14	28.18
		*t*-test	-	1.34e-3	1.76e-26	1.32e-4	5.75e-25	4.58e-11
	Angle	Mean	**0.08**	0.51	0.73	0.62	0.82	0.72
		Std. Dev.	**0.22**	0.47	0.28	0.46	0.31	0.30
	Time	Mean	5.46	6.14	**2.74**	2.87	3.52	2.77
M02	Path Length	Mean	**316.34**	318.69	320.25	317.71	319.47	319.86
		Std. Dev.	**2.03**	2.98	9.41	2.87	6.94	8.56
		*t*-test	-	7.92e-4	3.35e-2	3.79e-2	2.37e-2	3.59e-2
	Angle	Mean	1.20	1.71	1.22	**0.98**	0.99	1.07
		Std. Dev.	**0.25**	0.16	0.58	0.47	0.44	0.50
	Time	Mean	6.29	6.77	3.76	3.43	3.92	**3.09**
M03	Path Length	Mean	**299.94**	302.48	308.53	304.82	325.13	310.29
		Std. Dev.	**5.18**	7.16	12.15	9.49	13.54	8.19
		*t*-test	-	**1.22e-1**	9.81e-4	1.73e-2	1.60e-11	3.98e-7
	Angle	Mean	1.59	1.72	1.42	1.48	**1.22**	1.42
		Std. Dev.	**0.12**	0.17	0.30	0.25	0.43	0.27
	Time	Mean	6.22	6.57	3.64	**3.22**	4.17	3.51
M04	Path Length	Mean	**254.76**	261.10	264.76	259.26	268.69	278.38
		Std. Dev.	**7.34**	9.89	17.44	10.48	17.24	16.71
		*t*-test	-	6.71e-3	6.21e-3	**5.929e-2**	2.20e-4	1.45e-8
	Angle	Mean	1.57	1.90	1.51	**1.38**	1.43	1.53
		Std. Dev.	**0.25**	0.76	0.62	0.70	0.82	0.56
	Time	Mean	5.92	6.28	**3.26**	3.69	5.12	4.21
M05	Path Length	Mean	**270.13**	281.67	279.62	284.58	296.93	283.94
		Std. Dev.	**12.29**	22.96	24.28	18.20	20.21	19.97
		*t*-test	-	1.93e-2	**6.27e-2**	7.06e-4	1.22e-7	2.26e-3
	Angle	Mean	**1.18**	1.42	1.59	1.58	1.56	1.67
		Std. Dev.	**0.22**	0.29	0.34	0.36	0.38	0.43
	Time	Mean	5.71	6.29	4.14	**3.24**	4.56	3.72
M06	Path Length	Mean	**257.65**	273.41	293.69	293.59	284.20	286.96
		Std. Dev.	**1.91**	25.25	25.76	27.76	28.74	27.96
		*t*-test	-	1.92e-3	1.87e-8	8.35e-8	2.17e-5	3.26e-6
	Angle	Mean	**2.18**	2.41	2.14	2.48	2.65	2.70
		Std. Dev.	**0.50**	0.76	0.73	0.69	0.53	0.57
	Time	Mean	7.34	7.72	4.18	**3.55**	4.52	4.12
M07	Path Length	Mean	**321.99**	332.45	340.97	355.83	361.49	335.95
		Std. Dev.	**19.67**	50.16	37.69	31.20	41.76	49.91
		*t*-test	-	**2.94e-1**	1.86e-2	7.10e-6	3.01e-5	**1.62e-1**
	Angle	Mean	**3.20**	3.33	3.60	3.48	3.83	4.00
		Std. Dev.	**0.61**	1.49	1.28	1.17	1.21	1.20
	Time	Mean	5.41	5.87	**3.72**	4.15	4.52	5.51
M08	Path Length	Mean	**313.13**	325.94	333.84	329.88	318.64	326.12
		Std. Dev.	**15.76**	30.24	27.60	29.52	32.23	30.39
		*t*-test	-	4.57e-2	8.54e-4	8.81e-3	**4.06e-1**	4.37e-2
	Angle	Mean	**1.34**	1.56	1.85	1.59	1.63	1.75
		Std. Dev.	**0.17**	0.29	0.65	0.29	0.53	0.43
	Time	Mean	6.63	7.52	**3.72**	3.82	4.91	4.51
M09	Path Length	Mean	**315.84**	324.33	329.28	322.92	328.99	324.75
		Std. Dev.	**1.13**	19.86	13.24	11.27	9.39	12.20
		*t*-test	-	2.64e-2	5.35e-6	1.82e-3	1.77e-8	4.07e-4
	Angle	Mean	**1.33**	1.83	1.93	1.87	1.74	1.86
		Std. Dev.	**0.18**	0.40	0.47	0.52	0.53	0.45
	Time	Mean	5.15	5.62	3.71	**3.24**	4.79	4.53

It can be seen that in the nine test maps (Figs 3 and 5), the SSQL can effectively solve the path planning problem of MR from the starting position to the target position without collision with obstacles under short path length condition, and it can achieve excellent results (path length and turning angle) in majority of experiments except M02. The results demonstrate that the proposed algorithm effectively reduces the random motion in the initial phase of MR, further accelerating the convergence speed and reducing the computing time compared to the CQL. Our method achieves the optimal path length and turning angle in different environments, which demonstrates its practicality, generalizability, and robustness. Compared to the existing methods, the proposed approach can effectively escape from trap points due to local minima and find the optimal path. The limitation is that the computing time is longer compared to the comparison algorithms, but it is within the acceptable range.

To demonstrate the superiority of the proposed algorithm over the CQL algorithm in terms of path length turning angle and computing time. In this paper, the percentage improvement on the CQL algorithm is recorded in Tables 4 and 5. The results demonstrate that the proposed algorithm effectively reduces the random motion in the initial phase of MR, further accelerating the convergence speed and reducing the computing time.

**Table 4:** Comparison of algorithm performance between SSQL and CQL in short path planning.

	Path length vs CQL	Angle vs CQL	Time vs CQL
M01	7.06%	76.74%	9.48%
M02	1.24%	33.73%	10.02%
M03	1.25%	5.81%	12.63%
M04	1.79%	20.55%	11.60%
M05	2.80%	6.34%	5.22%
M06	1.31%	12.74%	6.91%
M07	4.54%	33.54%	5.21%
M08	3.87%	11.92%	7.59%
M09	1.57%	14.47%	3.15%

**Table 5:** Comparison of algorithm performance between SSQL and CQL in safe path planning.

	Path length vs CQL	Angle vs CQL	Time vs CQL
M01	9.22%	84.31%	53.19%
M02	0.74%	29.82%	7.09%
M03	0.84%	7.56%	5.33%
M04	2.43%	17.37%	5.73%
M05	4.10%	16.90%	9.22%
M06	5.76%	9.54%	4.92%
M07	3.15%	3.90%	7.84%
M08	3.93%	14.10%	11.84%
M09	2.62%	27.32%	8.36%

Test results under nine different cases validate that:

1. Our approach has the shortest path length and smallest turning angle of MR for short and safe path planning in all cases, compared with the comparison algorithms in this paper.
2. The proposed approach has the smallest standard deviations among the comparison algorithms in all cases, which demonstrates the superiority and stability of the DMQL algorithm under different environments.
3. From the time results in Tables 2 and 3, it can be seen that DSQL has improved compared to CQL, but the computing time slightly higher than PSO, GWO, DA and MFO.
4. The *t*-test results indicate that SSQL outperforms in all the cases because the values are smaller than the level 0.05 of significance.

In Figs 3A and 5A, we test the long-distance obstacle free path planning scheme. In Figs 3 and 5B–5F, we design the local minimum problem, which will cause the mobile robot to easily fall into the local optimum and cannot calculate the effective path. In Figs 3 and 5G–5I, we design the path planning problem under the maze and dense obstacles that may be encountered in the real environment. Through these maps and comparison algorithms, it can be proved that the proposed algorithm has the advantages of robustness, universality and wide application range. The experimental results show that the SSQL algorithm can enable the mobile robot to plan the path with the shortest distance and the smallest turning angle. The sub optimal algorithm is GWO, which performs well in most experiments, but its robustness and stability are not as good as SSQL. The performance of PSO is the worst in most experiments, which is specifically manifested in that the path is far away from the obstacles, resulting in the long path length, and the path is tortuous and the large turning angle. During the experiment, CQL cannot find the global optimal solution due to its slow convergence speed, resulting in the path tortuous, large turning angle and long path length. On the other hand, the biological heuristic algorithm is prone to premature convergence caused by falling into local optimization, while the principle of Q-learning algorithm leads to that the SSQL algorithm can finally find the global optimal solution under the condition that the time is long enough.

During the experiment, the mobile robot traverses the map to obtain the global information, and finds the optimal path (optimal path length and turning angle) in the process of continuous interaction with the environment. These results of path planning for MR show that: Our method achieves the optimal path length and turning angle in different environments, which demonstrates its practicality, generalizability, and robustness. PSO performs blind random search and cannot obtain the global map information, resulting in the inability to calculate the optimal or suboptimal solution, and lead to long computing time. DA introduces the initial population individual optimization mechanism and adds the local rationality judgment mechanism, which makes the algorithm easy to fall into the local minima when calculating the best result. GWO and MFO have achieved excellent results in the comparison algorithm, which is to explore the search space widely, but it still cannot find the global optimal solution like SSQL. Compared to the existing methods, the proposed approach can effectively escape from trap points due to local minima and find the optimal global path. The limitation is that the computing time is longer compared to the comparison algorithms, but it is within the acceptable range.

In the application of mobile robot path planning, different working modes can be adopted according to the size of the mobile robot, the density of obstacles in the environment and the actual needs. When the mobile robot aims to reach the target point as soon as possible, the short path planning mode of SSQL can be used to make the mobile robot reach the target with the shortest path length and turning angle. When the purpose of the mobile robot is to avoid collision with obstacles, the safe path mode can be adopted, and the mobile robot will plan the feasible path away from the obstacles. The combination of the two modes can be better applied in the working environment according to the actual use of mobile robots.

## Conclusions

In this paper, the SSQL for MR path planning algorithm is proposed, and simulation experiments are conducted according to the short and safe condition of MR in different environments. Two modes are switched for practical work, which can effectively solve the MR path planning problem, saving energy and time. By combining APF with the Q-learning algorithm to initialize the Q-table and setting the static and dynamic reward function to provide the prior knowledge in the environment to the MR, the convergence speed and computation time of CQL are accelerated.

To demonstrate the effectiveness and generality of the proposed algorithm, simulation experiments are set up for the maritime environments, fully considering the reefs around the coast in the actual work. It demonstrates the proposed SSQL algorithm can effectively solve the path planning problem for MR, and the mean, standard deviation and t-test of the data analysis show that the proposed algorithm yields the smallest path length and smoothness, which significantly speeds up the computation time and convergence speed of the CQL. The problem of MR falling into local minima and not being able to derive effective paths is avoided. The results show that the SSQL algorithm does not have precocity and can calculate the optimal solution, and it can be further seen that PSO, GWO, DA, and MFO are more prone to precocity in the environment with sparse obstacles.
